# Interventions for Hypertrophic Obstructive Cardiomyopathy: Defining the Gold Standard, Assessing Durability, and Guiding Patient Selection

**DOI:** 10.3390/medsci14010109

**Published:** 2026-02-24

**Authors:** Ajibola Anifowose, Marco Tagliafierro, Ghadeer Mahdi, Saada Hussein, Massimo Baudo, Tulio Caldonazo, Aleksander Dokollari, Kaveh Hosseini, William D. T. Kent, Ali Fatehi Hassanabad

**Affiliations:** 1 Faculty of Medicine and Dentistry, University of Alberta, Edmonton, AB T6G 2G5, Canada; 2 Section of Cardiac Surgery, Department of Surgery, Columbia University Irving Medical Center, New York, NY 10032, USA; 3 Division of Cardiac Surgery, Department of Cardiac Sciences, Libin Cardiovascular Institute, University of Calgary, Calgary, AB T2N 1N4, Canada; 4 Department of Cardiac Surgery Research, Lankenau Institute for Medical Research, Main Line Health, Wynnewood, PA 19096, USA; 5 Department of Cardiothoracic Surgery, Jena University Hospital, Friedrich Schiller University of Jena, 07747 Jena, Germany; 6 Section of Cardiac Surgery, St. Boniface Hospital, University of Manitoba, Winnipeg, MB R2H 2A6, Canada; 7 Department of Cardiology, Copenhagen University Hospital—Herlev and Gentofte, 2100 Copenhagen, Denmark

**Keywords:** hypertrophic obstructive cardiomyopathy, medical therapy, septal myectomy, septal ablation therapy, clinical outcomes

## Abstract

Hypertrophic obstructive cardiomyopathy (HOCM) is a genetic disorder characterized by a dynamic left ventricular outflow tract (LVOT) obstruction and an increased risk of sudden cardiac death. For patients with symptoms refractory to medical management, or intolerant to the new selective myosin inhibitor, septal reduction therapy (SRT) with surgical septal myectomy (SM) is indicated. This narrative review provides a contemporary assessment of septal myectomy, integrating its historical development, technical advancements, and comparative long-term outcomes. SM is established as the current reference standard, offering extensive anatomical relief and favorable long-term survival in clinical registries. It consistently achieves residual LVOT gradients <10 mmHg and enables correction of complex accessory mitral pathologies, leading to significant reverse ventricular and atrial remodeling. In contrast, ASA, a less invasive alternative for high-risk surgical candidates, is limited by incomplete tissue necrosis. This results in higher residual gradients (15–20 mmHg), increased rates of re-intervention (10–20%) and incidence of permanent pacemaker implantation (10–17.4%) and an associated greater risk of long-term all-cause mortality (>5 years). The disparity in long-term survival and the risk associated with sequential septal reduction procedures underscore the critical need for precise patient selection guided by institutional expertise. Furthermore, advancements like virtual surgical myectomy and transapical beating-heart septal myectomy are expanding the scope of intervention. This review synthesizes comparative contemporary data on HOCM management, highlighting the need for prospective, multicenter studies to address enduring knowledge gaps concerning equitable access, genotype integration, and long-term comparative effectiveness as current evidence remains dominated by retrospective studies.

## 1. Introduction

Hypertrophic obstructive cardiomyopathy (HOCM) is a genetic disorder characterized by asymmetric septal hypertrophy causing a dynamic left ventricular outflow tract (LVOT) obstruction [1]. This obstruction is most often driven by a combination of thickened septum and a systolic anterior motion of the mitral valve, ultimately giving rise to symptoms such as exertional dyspnea, angina, presyncope, syncope, and, in more severe cases, heart failure or sudden cardiac death (SCD) [2,3,4]. While the clinical course of HOCM is highly variable, untreated patients face an annual SCD incidence of 0.5–1.5% in adults and up to 2% in children and young adults [5]. Without intervention, these complications can evolve unpredictably with most HCM-related deaths occurring suddenly before age 60 [6], underscoring the necessity of early risk stratification and preventive therapy. Therefore, early HOCM recognition and treatment are pivotal to manage symptoms and life-threatening complications [3,4,7].

The underlying pathophysiology of HOCM is multifactorial and includes several structural changes, such as subaortic septal hypertrophy, mitral valve elongation, and abnormal papillary muscle anatomy. These elements drive LVOT obstruction, arrhythmias, ischemia, and mitral regurgitation [1,4,8]. Patient presentations with HOCM are heterogeneous, some may present with isolated symptoms or a unique interplay of multiple signs and symptoms. The severity of their symptoms is commonly assessed using the New York Heart Association (NYHA) functional classification, with many patients presenting in class III or IV by the time they are referred for advanced intervention [8,9].

Given the complex pathophysiology of HOCM and the spectrum of therapeutic options, this narrative review focuses on the role of septal myectomy (SM) as a reference intervention in comparison to alcohol septal ablation (ASA) as an alternative interventional treatment, including indications, technical considerations, patient outcomes, and complications, while situating the procedures within their historical and developmental context.

## 2. Methods

A comprehensive search of Ovid MEDLINE and PubMed was performed to identify publications regarding septal reduction therapies for HOCM. The search utilized MeSH terms and text words including ‘septal myectomy,’ ‘alcohol septal ablation,’ and ‘surgical techniques.’ Original research, systematic reviews, and meta-analyses published over the last three decades were prioritized, while animal studies and non-English articles were excluded. Landmark trials and foundational studies were manually examined to provide historical context. In synthesizing the clinical evidence, we screened for overlapping patient populations across publications; specifically, between primary trials (e.g., EXPLORER-HCM) and their subsequent long-term extensions analyses to ensure the narrative was not disproportionately weighted by duplicate cohorts.

## 3. Epidemiology and Clinical Burden

Hypertrophic cardiomyopathy (HCM) is the most common inherited heart disease, and HOCM represents the obstructive phenotype at rest and provocation, affecting two-thirds of HCM patients [2]. HOCM has a global prevalence, influenced by several factors that warrant consideration. This includes subclinical and clinically evident cases, as well as asymptomatic, unreported cases. In young adults, HCM incidence is approximately 1 in 500, including asymptomatic individuals [7]. When focusing on symptomatic HCM, medical data indicate a prevalence of less than 1 in 3000 adults in the United States [10]. Moreover, the diagnostic landscape is steadily evolving with advances in non-invasive cardiac imaging such as echocardiography and cardiac magnetic resonance (CMR). Both imaging modalities provide superior anatomic and functional detail, enabling more accurate characterization of ventricular wall anomalies and dynamic outflow tract gradients, ultimately leading to a better understanding of the disease burden.

Furthermore, HOCM is observed in populations with an equal distribution among male and female patients; however, studies indicate a higher level of diagnosis in females compared to males. Additional factors, such as ethnicity, may also influence the distribution of reported diagnoses, with findings depicting that patients of African descent diagnosed at younger ages are more likely to present with symptomatic heart failure (HF) and less likely to undergo genetic testing compared to their Caucasian counterparts [11]. Current evidence attributes these differences to sociodemographic factors and variations in their social determinants of health. These demographic disparities intersect with healthcare system structures, shaping referral patterns, diagnostic recognition, and access to definitive intervention. The inequities in the referral pathways, driven by social, geographic, and insurance-related factors, contribute to delayed management among minority and low-income populations. Consequently, Black patients, women, and Medicaid recipients in the United States are significantly less likely to receive advanced therapies such as septal reduction or ICD implantation and experience higher in-hospital mortality [12,13]. Compounding this issue, a major diagnostic barrier for Black patients is the frequent misattribution of left ventricular hypertrophy to systemic hypertension, further delaying appropriate clinical evaluation [14]. Collectively, these findings underscore that disparities in HOCM outcomes are strongly shaped by inequities in referral pathways, diagnostic recognition, and access to specialized care. However, the relative contribution of each factor remains incompletely defined and further epidemiological studies are necessary to better understand and characterize this phenomenon [1]. Importantly, these disparities exist within the context of substantial advances in HOCM management. In the modern era, improved management has reduced related SCD rates to 0.32% per year, though the risk remains significantly higher in pediatric populations (1.09% per year) [15]. Despite a 1.4–1.6-fold increased risk of all-cause mortality compared to the general population, most patients can now achieve near-normal longevity with appropriate, contemporary management [16].

## 4. Management of HOCM

The treatment options for HOCM are well-established and span both medical and procedural approaches. However, the treatment of choice is patient-oriented and depends on the symptomatology as well as surgical risk [8].

### 4.1. Pharmacologic Therapy

The first-line management for HOCM typically involves medical therapy. Beta-blockers, non-dihydropyridine calcium channel blockers, and disopyramide remain first-line agents for controlling patient symptoms, including dyspnea, angina, and exercise intolerance. These medications reduce myocardial contractility and slow the heart rate, which subsequently decreases the pressure gradient across the LVOT. However, these pharmacologic agents are not used for disease-modifying intent [1,2,3]. Additionally, newer pharmacological agents may be selectively employed in more nuanced patient cases before pursuing other procedural treatment options.

The emerging therapies, namely cardiac myosin inhibitors (e.g., mavacamten in adults), represent a newer therapeutic option that modulates cardiac contractility and relieves left ventricular outflow tract obstruction (LVOTO) by inhibiting the actin–myosin interaction within the cardiac muscle, thereby decreasing contractility and outflow obstruction. These agents have been validated and are guideline-recommended therapy shown to improve the LVOT gradients and functional capacity in 30% to 60% of patients with obstructive HCM. Furthermore, they are beneficial and can be indicated for treatment in patients with HOCM who do not receive adequate symptomatic relief (assessed through NYHA functional class measurements and pVO2 testing) despite the use of ≥1 first-line medical therapies [1,2,3,4,7,8,9,10,11,17]. In the multinational phase 3 EXPLORER-HCM trial, transient reductions in LVEF < 50% occurred in 7 of 123 patients (5.7%) treated with mavacamten compared with 2 of 128 patients (1.6%) receiving placebo. These events resolved with protocol-directed temporary interruption and did not preclude treatment completion, supporting the overall reversibility and manageable safety profile of the drug. While the therapy is validated by the U.S. FDA and endorsed as a Class 1 recommendation in the 2024 AHA/ACC guidelines, it requires careful monitoring. A thorough risk evaluation and mitigation strategy are essential, particularly given the observed decrease in LVEF and the potential risk of inducing heart failure [18,19].

### 4.2. Device Therapy

In other select cases, patients deemed at high risk of SCD are candidates for an Implantable Cardioverter–Defibrillator (ICD), which serves as a key preventive measure [1,20]. The indication for this device therapy is strongly emphasized particularly following instances of SCD in competitive athletes, which is the most prominent complication of HCM regardless of LVOTO.

To guide this risk stratification, a collaboration between the American College of Cardiology (ACC) and the American Heart Association (AHA) on the consensus management of HOCM developed an algorithm detailing the major markers that serve to identify patients at risk (defined as ≥ 1 marker) of SCD [1,21,22]. These markers that strongly support primary prevention with ICD placement include: a history of cardiac arrest or sustained ventricular tachycardia (VT) (secondary prevention), unexplained syncope likely arrhythmic in origin, a family history of HCM-related SCD in a first-degree relative ≤ 50 years of age, left ventricular hypertrophy (LVH ≥ 30 mm), a left ventricular (LV) apical aneurysm, systolic dysfunction (LVEF < 50%), and repetitive Non-Sustained VT (NSVT). These primary risk factors are supplemented by risk mediators which on their own may not warrant ICD placement but are used by clinicians to assess overall risk. These include extensive late gadolinium enhancement (LGE ≥ 15% of LV mass) on CMR, significant LVOTO at rest (≥50 mm Hg), an abnormal hypotensive response to exertion during cardiopulmonary exercise testing (CPET), and a prior history of ASA. These comprehensive assessment markers ensure that the ICD therapy is reserved for those with the highest demonstrated risk profile [1].

### 4.3. Cardiac Pacing: Specialized Therapeutic Adjunct for Non-Surgical Candidates

For patients with drug-refractory HOCM who remain symptomatic despite medical therapy but are not candidates for septal reduction therapy, cardiac pacing has been evaluated as a device-based adjunct for symptom relief. Dual-chamber pacing (DDD) with a right ventricular (RV) apical lead and optimized atrioventricular (AV) delay can acutely reduce the LVOT gradient by altering the sequence of septal activation [23]. While contemporary clinical practice guidelines acknowledge this as a Class IIb consideration for symptomatic relief in select older patients or those with mild septal hypertrophy [1,24], RCTs have demonstrated only modest long-term benefits in functional capacity. For instance, the EMORI-HCM trial observed an 18% reduction in resting gradients and a marginal increase in peak oxygen uptake, effects that remain less pronounced than those achieved with contemporary myosin inhibition (Mavacamten) [23].

As randomized trials have shown only modest functional improvement with DDD pacing, subsequent studies have examined whether BiV or LV pacing might provide more sustained hemodynamic benefit and reverse remodeling, although the supporting evidence remains limited and largely non-randomized. Pilot studies of biventricular (BiV) or left ventricular (LV) pacing have suggested progressive reductions in LV mass and LVOT gradients over one year [25]; however, these findings are from small, non-randomized cohorts and lack confirmation in larger studies. Importantly, standard cardiac resynchronization therapy (CRT) is generally reserved for patients with established heart failure indications, such as reduced LVEF and LBBB, rather than for gradient reduction alone [1]. Consequently, the current consensus does not strongly support routine pacing for disease modification or survival benefit, positioning it as a highly individualized option for symptomatic management in specialized cases.

### 4.4. Invasive Treatment: Septal Reduction Therapies

Besides pharmacological and device therapy options, advanced interventions such as surgical myectomy or alcohol septal ablation are often considered in patients who exhibit sub-optimal therapeutic response or have no medical contraindications. The need for such therapy is significant; the SHaRE registry, which tracks 10,225 HCM patients, found that approximately 18% of patients required septal reduction therapy [26]. Surgical septal myectomy and alcohol septal ablation are indicated for patients with persistent symptoms despite optimal medical therapy and a significant LVOT gradient (≥50 mmHg). These procedures are endorsed by both the ACC and AHA as standard interventions for drug-refractory cases [1].

## 5. Septal Myectomy: The Gold Standard

Septal myectomy, first introduced by Dr. Andrew Glenn Morrow in the early 1960s [27], remains the gold standard for septal reduction in patients with severe, drug-refractory symptoms and significant LVOT gradients with other associated cardiac disease necessitating surgery (i.e., anomalous papillary muscle, intrinsic mitral valve disease). The original approach involved open-heart resection of the hypertrophied septum via a transaortic route with a median sternotomy. Over time, the procedure has evolved as the mainstay approach including an extended myectomy, adjunctive mitral valve and subvalvular interventions, and, more recently, minimally invasive and video-assisted techniques. The transaortic extended septal myectomy (ESM) approach is highly effective with clinical success > 90% and a mortality rate of <1% at experienced tertiary centers [1]. These refinements have enhanced procedural safety and efficacy while expanding the range of patients who may benefit from surgery.

### 5.1. Indications and Contraindications

The surgical procedure is recommended for patients with symptomatic HOCM exhibiting suboptimal response to medical therapy. Candidates typically have an LVOT gradient of ≥50 mmHg at rest or with provocation, alongside severe symptoms (NYHA class III or IV) [19]. In some cases, early surgical intervention may be considered for younger patients with a high risk of SCD or significant symptom burden. The ideal surgical candidate presents with significant obstruction, disabling symptoms, and no prohibitive comorbidities and patients with preserved systolic function and favorable anatomy benefit most from this intervention [1,3,16,28,29].

The criteria for septal myectomy candidacy are summarized in Table 1. The contraindications to septal myectomy typically involve patients presenting with prohibitive surgical risk factors, such as advanced age and overall frailty, as well as the presence of severe comorbid conditions, including advanced pulmonary disease, severe renal dysfunction, unstable coronary artery disease, poorly controlled hypertension, and complicated diabetes. Additionally, patients with non-obstructive HCM with mild or controlled symptoms or having a favorable response to pharmacologic therapy are generally not considered for surgery [1,3,16,28,29,30].

### 5.2. Standard Procedural Techniques and Adjunctive Interventions

The procedure is technically demanding and is typically performed at specialized HCM centers, as recommended by the ACC and AHA. The standard approach involves general anesthesia and cardiopulmonary bypass to ensure a motionless, bloodless field for precise resection. The most common method used to perform the procedure is via a median sternotomy and transaortic access, allowing for direct visualization of the hypertrophied basal septum [4,17,31].

The extended myectomy involves resecting the septal myocardium from just beneath the aortic valve to the level of the papillary muscles. The resection is tailored to the extent and location of the hypertrophy in the heart. During the procedure, intraoperative transesophageal echocardiography is essential in guiding the extent of the resection and confirming the resolution of the LVOTO and mitral regurgitation [4,8,17,31]. A transapical approach may be preferred, or used as an adjunct to the transaortic route due to anatomical indications, such as midventricular or apical hypertrophy. This is relevant for extensive apical disease or in pediatric patients with a small aortic annulus.

Furthermore, in patients with mitral or subvalvular abnormalities that contribute to additional obstruction or systolic anterior motion (SAM) of the mitral valve leaflets, adjunctive procedures such as papillary muscle release and anterior mitral leaflet plication are performed during the septal myectomy procedure. At experienced centers, concurrent cardiac procedures, such as mitral valve repair or coronary artery bypass grafting can also be safely integrated into the operation without increasing risk for the patient [1,4,17,32].

### 5.3. Outcomes and Long-Term Durability

Septal myectomy has evolved into a safe and effective surgical intervention for patients with HOCM, particularly when performed in experienced centers and with advanced cardiac imaging techniques for better characterization.

#### 5.3.1. Early Operative Risk and Procedural Safety

Historically, SM was associated with substantial procedural risks, including early mortality rates ranging from 5% to 10% and a high incidence of major complications such as complete heart block, aortic regurgitation, and ventricular septal defect (VSD) [33]. However, contemporary outcomes have markedly improved in high-volume, specialized HCM centers. Across multiple studies, findings concluded that when performed by experienced surgical teams, an isolated septal myectomy procedure now carries a mortality risk of less than 1% [17]. The rate of complete heart block necessitating PPM implantation has dropped to <3%, while other major perioperative complications occur in <1% of patient cases. The hemodynamic post-surgery outcomes are also favorable, with most patients achieving a residual LVOT gradient reduced from 50 mmHg to less than 10 mmHg, even under provocation with exercise [17].

#### 5.3.2. Real-World and Volume-Dependent Variation in Outcomes

Despite these advances in surgical outcomes and complication rates, U.S. nation-wide inpatient data suggest that surgical outcomes can vary widely based on institutional experience. National registry data from 2003 to 2011 show real-world hospital mortality for septal myectomy ranging from 4–16%, in sharp contrast to the <1% operative mortality reported by high-volume expert centers [34]. Similarly, the rates of procedural complications such as VSD and inadequate relief of LVOT obstruction are significantly higher in low-volume or less experienced institutions. The Society of Thoracic Surgeons Adult Cardiac Surgery Database from 2012–2019 revealed that centers performing 1 to 5 septal myectomy cases annually had a mortality rate of 3.7% and a VSD incidence of 2.6%, compared with <1% mortality and a 0.4% VSD rate in centers performing ≥10 myectomies per year [35]. These findings have underscored the importance of surgical volume and institutional expertise in determining favorable patient outcomes.

In recognition of this, the ACC/AHA Guidelines recommended for septal reduction therapies to be conducted only in specialized centers with experienced operators and integrated HCM programs, aiming for an operative mortality rate of less than 1% and a major complication rate of under 3% [1,2].

#### 5.3.3. Symptom Relief and Functional Improvement

A successful myectomy intervention results in substantial and durable symptom relief for patients who undergo the procedure. Most patients, approximately 80% to 90%, are classified as New York Heart Association (NYHA) class III–IV pre-surgery and I–II following surgery, reporting significant improvements in exertional dyspnea, angina, and syncope [4,8,17,20,36]. Importantly, patients with latent or provocable obstruction benefit similarly to those with severe resting obstruction. Furthermore, in most instances, long-term symptom recurrence is unrelated to muscle regrowth but rather attributable to inadequate resection at the time of the initial procedure or the development of a midventricular obstruction [37].

#### 5.3.4. Long-Term Outcomes and Survival

Longitudinal research data depicts the lasting benefits and anatomical durability of the septal myectomy procedure. Surgical patients experience sustained improvements in both their subjective symptoms and objective measures, such as exercise capacity with follow-up studies consistently indicating no evidence of recurrent obstruction caused by septal muscle regrowth.

The survival outcomes after the procedure are highly favorable, with reported 5-year survival rates ranging from 84% to 96%, while 10-year survival rates span 71% to 88%. Specific large cohort studies have demonstrated this durability with survival rates of 88% at 10 years and 72% at 20 years reported following the procedure [38]. Similarly, survival rates were 83% at 10 years and 68% at 20 years in another cohort of 338 adult patients [39]. One study further reported 10-year survival rate of 83% among 289 patients, a rate comparable to that of an age- and sex-matched general U.S. population, as well as to HCM patients without obstruction [40]. Notably, survival is higher among patients undergoing isolated myectomy compared to patients undergoing combined procedures [41]. Furthermore, about 25% of HCM patients ultimately die from their disease, with HCM-related mortality being highest among individuals under 30 years of age [42].

### 5.4. Complications and Management

The complications following septal myectomy are infrequent at high-volume centers; however timely recognition and appropriate management are essential. While surgical myectomy is considered a low-risk, high-benefit treatment, it still entails an open-heart procedure with sternotomy. Thus, this surgical technique presents with 30-day adverse complications such as tamponade or an LAD injury [1]. Additional common post-operation complications include bleeding, infection, cardiac arrhythmias and conduction abnormalities, such as a left bundle branch block (LBBB) or complete heart block requiring pacemaker implantation [1,17,20,31,36,41,43].

Intraoperative bleeding is managed through surgical hemostasis and adjunct antifibrinolytics, while postoperative bleeding may require transfusion or re-exploration [44]. The risk of infection with the procedure is minimized through perioperative antibiotic use and sterile precautions. Additionally, deep sternal wound infections, though rare, do require prompt surgical and antimicrobial treatment [4,31].

Arrhythmias are also a notable concern and a common complication of HCM, leading to a significant increase in the risk of a thromboembolic stroke event. In patients with postoperative atrial fibrillation, they are managed with rate or rhythm control strategies, anticoagulation therapy, and, in select cases, intraoperative interventions such as pulmonary vein isolation or a maze procedure [45]. Conversely, while ventricular tachycardia is a less common presentation, when present it is addressed with antiarrhythmic therapy or ICD [1,3,17,20,43].

Within the population of pregnant patients with HCM, certain complications often arise during the third trimester and may include heart failure, dyspnea, chest pain, and palpitations. These occur due to the significant hemodynamic changes of pregnancy and the associated strain on the cardiovascular system, which can exacerbate the underlying pathology of HCM. They are often treated with medical therapies such as beta-blockers and low molecular weight heparin (LMWH) with close monitoring of fetal growth. Commonly, vaginal delivery is the first-line choice of delivery, with a subsequent consultation and follow-up with cardiologist and obstetrician [1,46,47,48].

Lastly, conduction disturbances are a well-recognized complication in patients with HCM. Specifically, LBBB is observed in about 39% of cases, while complete heart block occurs in approximately 2.3% of patients, predominantly in individuals with a pre-existing right bundle branch block. The use of PPM implantation is further indicated in patients with a persistent complete heart block. Additionally, the need for rhythm pacing in HCM has been associated with increased long-term mortality, underscoring the need for careful risk stratification and ongoing follow-up in this patient population [1,43].

## 6. Emerging Surgical Techniques and Preoperative Planning

While surgical septal myectomy remains the gold-standard treatment for patients with drug-refractory HOCM, recent technological innovations are targeted towards refining the procedure to be less invasive, more precise, and better tailored to individual patient anatomy [49].

### 6.1. Advanced Preoperative Planning

Beyond the operating room, advances in preoperative planning are transforming how surgeons approach myectomy. Virtual surgical myectomy, utilizing patient-specific cardiac magnetic resonance (CMR) imaging, enables the creation of 3D models of the patient’s left ventricle. With the aid of specialized software, surgeons can visualize the precise location and extent of hypertrophy to perform a virtual resection on the model. The simulation is conducted during the end-diastolic phase to mimic the heart’s state during surgery, providing dexterous control and optimizing the surgical plan to avoid excessive resection or damage to adjacent structures. In a study of five patients, this virtual planning demonstrated a significant increase in stroke volume after the simulation myectomy, highlighting its potential to predict surgical outcomes and improve procedural success [49].

### 6.2. Novel Surgical Techniques

A significant development is the transapical beating-heart septal myectomy (TA-BSM), a minimally invasive procedure designed to simplify septal reduction therapy. The procedure is performed through a mini-thoracotomy without cardiopulmonary bypass with the use of the Beating-Heart Myectomy Device (BMD) under real-time echocardiographic guidance. It enables the surgeons to perform repeated resections with the opportunity to immediately evaluate the results on the beating heart, thereby tailoring the myectomy to effectively eliminate the LVOT obstruction and associated mitral regurgitation concurrently. Published in 2023, a first-in-human trial involving 47 patients demonstrated high procedural success, with 42 of 46 patients meeting the primary outcome at 3 months [50]. The maximal median LVOT gradient of the 47 participants was substantially reduced from a baseline of 86 mmHg to 19 mmHg. This approach offered the dual benefits of reducing surgical trauma while enabling real-time, guided resection. However, there were reported major adverse events, including one ventricular septal perforation and one apical tear [50].

Concurrent robotic-assisted surgery advances are also being made in minimally invasive approaches for septal myectomy. This approach, which often utilizes a trans-mitral route via a minimally invasive thoracotomy and left atriotomy, not only offers cosmetic advantages but also facilitates the surgical correction of concurrent mitral valve pathologies [51]. The precision of this technique is crucial, as the extent of tissue removal must be sufficient to relieve obstruction without causing iatrogenic complications like ventricular septal defects or heart block. A key innovation in this approach is the integration of intracardiac ultrasonography during the procedure facilitating real-time intraoperative assessment of the myocardium before and after resection, to enhance the safety and accuracy of the myectomy [51].

## 7. Alcohol Septal Ablation: A Key Alternative

### 7.1. Historical Context, Mechanism, and Indications

In contrast to the “gold standard” Septal Myectomy procedure, Alcohol Septal Ablation (ASA), initially introduced in 1995 by Ulrich Sigwart, provided a highly anticipated, less invasive, catheter-based approach for septal reduction in patients [7,52].

ASA achieves its therapeutic effect through a process of controlled septal infarction, resulting in myocardial necrosis, subsequent scar formation, and eventual tissue remodeling and shrinkage in the basal interventricular septum [2,28]. This mechanism results in a gradual relief of the obstruction, with significant hemodynamic and symptomatic improvements typically requiring 3–6 months to evolve fully [2,17]. Contemporary practice guidelines position ASA as an acceptable alternative (Class I) for symptomatic HOCM patients who are ineligible for SM or present with prohibitive surgical risk [1,31]. The literature consistently indicates that ASA is favored for older patients and/or those with significant comorbidities or frailty [20,29,31]. While SM is widely applicable across age groups, ASA is specifically discouraged in younger patients, typically those under 40 years of age, due to concerns regarding long-term outcome durability and the higher likelihood of requiring subsequent interventions [1,2].

### 7.2. Procedural Technique

The procedure mandates careful technique to ensure its safety and efficacy. The key steps include the insertion of a temporary right ventricular pacemaker lead, precise identification of a suitable septal perforator coronary artery, and advancing an over-the-wire balloon catheter into the target vessel [28]. Precision is crucial due to reliance on the myocardial contrast echocardiography (MCE) to confirm that the selected septal branch supplies the precise area of the septum involved in the SAM and mitral-septal contact [1,2,28]. Once the target is verified and vessel spillage is prevented by balloon inflation, a small volume of desiccated alcohol is injected slowly to induce the localized infarct [28]. Pressure monitoring, involving the simultaneous measurement of the LV apex and aortic pressures, is essential both before and after alcohol delivery to quantify the trans-septal gradient relief [1,2,28]. The patients require extended cardiac telemetry post-procedure to monitor for any conduction abnormalities that occur post-operation [29].

### 7.3. Outcomes, Complications, and Comparison

Both ASA and SM demonstrate established efficacy in relieving obstruction and providing symptomatic relief, with reports showing that over 90% of patients experience symptomatic improvement (reduction of ≥1 NYHA functional class) in experienced centers [1,17,31]. Furthermore, when performed at high-volume centers, the early (30-day) mortality rate for ASA is low, typically reported as <1% [1,2,17,31,52].

#### 7.3.1. Efficacy and Gradient Reduction

A major functional distinction between ASA and SM lies in the completeness of the gradient reduction. While SM typically achieves residual resting LVOT gradients of <10 mmHg, the typical residual LVOT gradient post-ASA is higher, frequently landing in the range of 15–20 mmHg [2,52,53]. The difference in gradient reduction between the two procedures stems from the fundamental limitations of ASA. The level of obstruction relief achieved by ASA is constrained by the infarct size that can be obtained based on the available coronary vessel anatomy. This is less effective than SM, where the surgeon can operate with direct visualization to resect all the necessary obstructing tissue precisely. This lack of anatomical completeness with ASA is what leads to higher failure rates, clinically evidenced by the elevated rates of re-intervention required after ASA procedures. More specifically, repeat procedures (either a second ASA or subsequent SM) are required in 10% to 20% of patients after an initial ASA procedure [17,52,54]. Propensity-matched data have emphasized this difference, and highlight a significantly increased risk for re-intervention following ASA compared to SM (Hazard Ratio, 33.3) [53].

#### 7.3.2. Procedure-Specific Complications

The most prominent complication associated with ASA is the rate of high-degree atrioventricular block requiring PPM implantation. Research studies consistently report a post-ASA PPM rate ranging from 10% to 17.4% [1,2,43,52,55]. This is notably higher than the rate achieved in dedicated SM centers, which typically report a PPM rate of <5% [1,43,55].

#### 7.3.3. Long-Term Survival

Regarding overall mortality outcomes, historical meta-analyses have suggested similar long-term survival between ASA and SM in selected cohorts [52,55]. However, contemporary larger cohort studies and recent meta-analyses focusing on extended follow-up periods (>5 years) challenge this equivalence, presenting that ASA is independently associated with an increased risk of long-term all-cause mortality compared to SM [1,52,56]. This may be attributable not only to patient selection bias with ASA being reserved for high surgical risk and frail patients but also to procedure-related factors such as the creation of a potentially arrhythmogenic infarct scar, higher residual LVOT gradients, and the adverse long-term effects associated with ventricular paced rhythm [1,56].

### 7.4. Limitations and Anatomical Flexibility

ASA is not considered the “gold standard” because SM offers a more durable, complete, and anatomically flexible relief, leading to superior long-term survival outcomes [1,2,17,52,55,56]. The gradient reduction for the LVOT is sometimes less complete after ASA primarily due to the reliance on a favorable septal perforator anatomy [17]. If the target area corresponding to the SAM-septal contact point is not adequately supplied by a suitable coronary vessel, the procedure may fail to relieve the obstruction completely in the patient [2].

Unlike SM, ASA cannot address a complex LV chamber morphology or any co-existing structural primary pathologies of the mitral valve (MV) or submitral apparatus (e.g., elongated leaflets, anomalous papillary muscles) that contribute to a high LVOT gradient. Performing septal myectomy with direct visualization enables the surgeon to tailor the resection and address the accessory obstructive components within the patient’s heart [1,2,16,17,28].

### 7.5. Implications of Sequential Septal Reduction Therapy

The evidence regarding long-term outcomes strongly favors SM, as it offers long-term survival comparable to that of the age- and gender-matched general population [52]. Conversely, when redo SM surgery is performed following a failed ASA, outcomes are compromised, primarily due to the conduction tissue damage induced by the initial alcohol infarct [43,53]. The permanent septal scar from ASA increases the risk of inducing complete heart block (CHB) during the subsequent myectomy, leading to a notably higher rate of PPM implantation compared to isolated SM [43,56]. This inherent risk associated with sequential septal reduction interventions highlights why SM is the more definitive and anatomically comprehensive initial therapy for most eligible patients.

## 8. Septal Myectomy Versus Alcohol Septal Ablation

This section synthesizes the key findings from this review, leveraging the detailed outcomes discussed in Section 5.3 and Section 7.3. A structural comparison of the two primary interventional strategies is summarized in Table 2. Both septal reduction therapies demonstrate high rates of symptomatic improvement (reduction of ≥1 NYHA functional class) in patients with drug-refractory HOCM. However, the procedures diverge significantly in their mechanism, resulting in critical differences in anatomical completeness, long-term durability, and complication profiles in patients.

### 8.1. Head-to-Head Comparison of Outcome

SM remains the gold standard because it offers the definitive, surgically tailored relief, leading to superior and more sustained hemodynamic results in patients. A significant distinction lies in the completeness of the gradient reduction. While SM typically achieves residual resting LVOT gradients of <10 mmHg, the typical residual gradient post-ASA is higher, frequently landing in the range of 15–20 mmHg. ASA’s reliance on localized tissue necrosis can lead to higher failure rates, as evidenced by the significantly elevated rate of re-intervention required in 10% to 20% of patients, a risk starkly emphasized by propensity-matched data (Hazard Ratio, 33.3).

Crucially, SM also offers superior anatomical flexibility, as it is performed under direct visualization, allowing the surgeon to address complex LV chamber morphology and co-existing structural issues of the mitral valve or submitral apparatus that contribute to the LVOT gradient. The inability of ASA to address these accessory obstructive components is a primary reason for less complete relief of the gradient. Another area of comparison lies in the functional and morphological reversal, patients successfully treated with SM often exhibit post-procedural regression of left ventricular hypertrophy (IVS thickness reduction from 2.05 ± 0.43 cm to 1.32 ± 0.34 cm at follow-up) and reverse remodeling of the left atrium (median left atrial volume index [LAVI] reduction from 48 mL/m^2^ to 38 mL/m^2^ at 2 years). This reverse remodeling, including the significant reduction in LV mass index, continues during the first two years after the operation and is attributed to a sustained LVOT gradient relief.

In addition, procedure-specific complications also strongly favor SM over ASA in experienced centers. The most prominent complication of ASA is the rate of high-degree atrioventricular block requiring PPM, consistently reported between 10% and 17.4%. This is notably higher than the rate achieved in dedicated SM centers, which is typically <5%.

### 8.2. Long-Term Survival and Evidence Limitations

The evidence base comparing the two interventions is inherently limited by the predominance of retrospective designs and the lack of randomized controlled trials (RCTs) comparing SM directly with ASA. Furthermore, when redo surgery (SM) is performed after a failed ASA, the outcomes are compromised due to the conduction tissue damage induced by the initial alcohol infarct, resulting in a higher rate of PPM implantation compared to isolated SM. Consequently, the clinical superiority of SM over ASA observed in large registries may be influenced by a residual confounding regarding the clinical profiles of patients selected for each intervention differing across centers.

## 9. Cardiac Myosin Inhibitors: An Evolving Landscape in the Management of HOCM

The longitudinal evidence for myosin inhibition, established from the MAVA-LTE (the long-term extension of EXPLORER-HCM) and VALOR-HCM trials, demonstrates that the hemodynamic improvements achieved with mavacamten are sustained over the long-term [1,57,58]. Over 180 weeks of follow-up, mavacamten therapy provided durable disease modification characterized by significant, lasting reductions in resting and provoked LVOT gradients, and myocardial stress biomarkers, such as NT-proBNP [57]. Furthermore, beyond physiological relief, mavacamten facilitates favorable reverse cardiac remodeling in patients, by reducing left ventricular mass and left atrial volume index; both critical predictors of adverse outcomes in the obstructive phenotype [57]. This durability does translate into meaningful functional gains, with nearly 80% of patients maintaining an improvement of ≥1 NYHA functional class over multiple years of treatment [59].

The clinical positioning of myosin inhibitors has transitioned from a secondary medical option to a viable upstream alternative to invasive SRT. VALOR-HCM data indicate that most patients meeting eligibility for SRT avoided these procedures after initiating mavacamten, with <16% requiring SRT by week 128 [58]. This shift suggests that early pharmacological intervention can defer the need for invasive reduction therapies in appropriately selected symptomatic patients. However, it is essential to view these therapies as alternatives rather than adjunctive strategies as currently, no clinical evidence supports the combination of myosin inhibitors with SM or ASA for HOCM management [58,60]. In contemporary practice, CMIs may serve as a bridge for patients who remain symptomatic on first-line pharmacological agents but wish to defer or avoid invasive SRT, effectively shifting their management course toward earlier, non-invasive disease modification.

**Figure 1:**
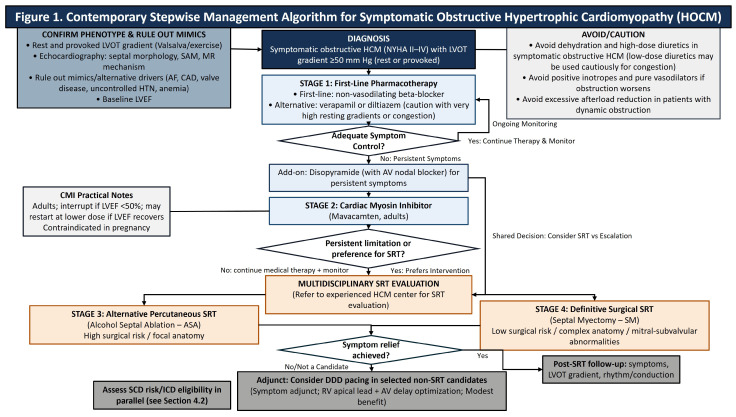
Contemporary stepwise management algorithm for symptomatic obstructive HOCM. LVOT gradients are peak instantaneous Doppler-derived gradients (rest or provoked); clinically significant obstruction is typically LVOT gradient ≥50 mm Hg in symptomatic patients. SCD risk stratification/ICD consideration should occur in parallel (see Section 4.2). Mavacamten (adults) requires serial LVEF monitoring and interruption if LVEF <50% and is contraindicated in pregnancy. Abbreviations: AF, atrial fibrillation; ASA, alcohol septal ablation; AV, atrioventricular; CAD, coronary artery disease; CMI, cardiac myosin inhibitor; HCM, hypertrophic cardiomyopathy; HTN, hypertension; ICD, implantable cardioverter–defibrillator; LVEF, left ventricular ejection fraction; LVOT, left ventricular outflow tract; MR, mitral regurgitation; SAM, systolic anterior motion; SCD, sudden cardiac death; SM, septal myectomy; SRT, septal reduction therapy.

The effective implementation of mavacamten requires a stringent, imaging-guided safety framework to manage the risk of systolic dysfunction [61]. The FDA-mandated monitoring protocol, necessitated by the drug’s mechanism of reducing contractility, requires a baseline LVEF > 55% and serial echocardiographic assessments to ensure the LVEF remains above the 50% interruption threshold [61]. While approximately 8% to 14% of patients may experience transient declines in LVEF < 50%, trial data confirms that these events are typically reversible upon protocol-directed dose interruption [57,58]. Furthermore, although recent regulatory updates have streamlined these monitoring requirements, vigilant surveillance is imperative to balance therapeutic efficacy against the risk of drug-induced HF. Clinicians should also be aware of specific contraindications, most notably in pregnancy, as mavacamten has teratogenic risks identified in preclinical studies, requiring effective contraception for women of childbearing potential [61]. To translate these therapeutic considerations into a bedside-oriented framework, Figure 1 summarizes a stepwise approach to the evaluation and management of symptomatic HOCM, integrating pharmacotherapy, cardiac myosin inhibition, septal reduction strategies, and selected device-based adjuncts.

## 10. Key Knowledge Gaps and Future Research Priorities

The literature review identifies three main themes that should be considered as priorities for ongoing and future studies: durable relief and predictable outcomes, technical innovation and integration, and comparative effectiveness and equitable access to definitive care. While SM remains the standard for durable relief of obstruction, the key knowledge gaps include the need for well-designed prospective studies that evaluate the optimal patient selection, especially in borderline cases or patients with complex anatomy. Secondly, the emerging surgical approaches (TA-BSM, robotics) and advanced preoperative planning require methodical evaluation of their durability, safety, and learning curves to define their role alongside the conventional extended transaortic myectomy. There are also still evidence gaps that persist, including the long-term comparative effectiveness and equitable access to definitive care. The focus should include prospective comparisons with standardized endpoints, long-term follow-up beyond ten years, and better characterization of selection criteria for complex anatomy. Additionally, the integration and consideration of patients’ genetic factors, as well as novel pharmacological approaches such as myosin inhibition, should further refine surgical decision pathways. Within epidemiologic research on HOCM, the gap in underdiagnosis and differential access, particularly across ethnic lines, highlights the importance of equity that should be addressed through inclusive trials and referral pathways that broaden access to specialized centers.

## 11. Conclusions

The current evidence strongly establishes septal myectomy as the primary surgical intervention for drug-refractory HOCM, given its association with anatomical completeness, improved long-term survival comparable to the general population, and capacity to induce beneficial reverse remodeling. While alcohol septal ablation provides a less invasive alternative for high-risk surgical candidates, its fundamental reliance on limited cardiac tissue necrosis results in less complete LVOT gradient reduction and carries a significantly higher risk of re-intervention and permanent pacemaker implantation. The field is expanding through technical innovation with virtual surgical planning and the Transapical Beating-Heart Septal Myectomy approach aimed at refining the precision and expanding patient candidacy. However, the evidence base remains limited by the lack of prospective, randomized trials comparing septal myectomy and alcohol septal ablation. This highlights the need for high-quality, multicenter studies that incorporate novel pharmacology and assess the long-term survival beyond ten years for patients, to ensure equitable access to definitive specialized care.

## Figures and Tables

**Table 1:** Eligibility and Selection Criteria for Septal Myectomy in Obstructive HCM.

Category	Criteria
**I. General Eligibility (Class I Indication)**	
Symptom status	Severe, drug-refractory symptoms (dyspnea, chest pain, presyncope/syncope) affecting quality of life. Despite optimal medical therapy.
Functional status	Typically, NYHA Class III–IV [8,9]. May be considered in select Class II patients at experienced centers.
**II. Hemodynamic and Anatomical Requirements**	
Hemodynamic threshold	Dynamic LVOT gradient ≥ **50** mmHg at rest or provocation (e.g., Valsalva maneuver) [19].
LVOT mechanism	Basal septal hypertrophy with systolic anterior motion (SAM) of the mitral valve.
Septal morphology	Adequate septal thickness to permit safe resection. Most appropriate when septal thickness ≥ **30** mm [1].
**III. Indications favoring septal myectomy over ASA**	
Need for concomitant surgery	When valve repair/replacement or other cardiac surgery is planned (single-procedure advantage).
Complex anatomy unsuitable for ASA	Midventricular obstruction. Apical hypertrophy (including cases requiring transapical access). Anomalous papillary muscles, accessory muscle bundles, or chordal abnormalities. Fixed subaortic obstruction (e.g., subaortic membrane).
Age and patient profile	Preferred in younger patients or those with marked hypertrophy.

Summary of guideline-based criteria and expert consensus indications for surgical myectomy. Abbreviations: ASA, alcohol septal ablation; HCM, hypertrophic cardiomyopathy; LVOT, left ventricular outflow tract; NYHA, New York Heart Association; SAM, systolic anterior motion.

**Table 2:** Comparative Features of Septal Reduction Strategies in Obstructive Hypertrophic Cardiomyopathy.

Category	Septal Myectomy (SM)	Alcohol Septal Ablation
Indication/patient profile	First-line therapy for eligible patients. Favored in younger patients or those with long expected survival.	Alternative for patients who are poor surgical candidates (e.g., advanced age, frailty, multimorbidity).
Procedure type	Open-heart surgery requiring median sternotomy and cardiopulmonary bypass.	Percutaneous catheter-based septal branch alcohol infusion.
Mechanism of relief	Direct excision of hypertrophied septum.	Alcohol-induced myocardial infarction with secondary scar-mediated septal thinning.
LVOT gradient reduction	Typically, <10 mmHg residual gradient in experienced centers [17].	Less complete (commonly 15–20 mmHg residual gradient) [2,52,53].
Reverse LV/LA remodeling	Consistent improvement in LVH and LA volume reduction.	Variable and often absent due to replacement of tissue with infarct scar.
Ability to address accessory anatomy	Yes: enables correction of mitral/subvalvular abnormalities (e.g., papillary muscle reorientation, chordal release).	No: limited to septal perforator anatomy; cannot address accessory mitral or subvalvular pathology.
Risk of permanent pacemaker (ppm) placement	Low (<5% in experienced centers) [17].	Higher (10–17.4%) [53,54].
Re-intervention rate	Low (<1%).	Higher (10–20% require repeat intervention) [17,52,54].
Long-term survival	Comparable to age- and sex-matched general population in contemporary series [16,20].	Some studies suggest possible association with increased long-term all-cause mortality [52,56].

Comparison of procedural and long-term outcomes for SM vs. ASA. Abbreviations: ASA, alcohol septal ablation; LA, left atrium; LV, left ventricle; LVH, left ventricular hypertrophy; LVOT, left ventricular outflow tract; SM, septal myectomy.

## Data Availability

No new data were created or analyzed in this study.
